# MR Imaging of Pulmonary Surfactant Distribution in a Preclinical Neonatal Lung Model

**DOI:** 10.1002/nbm.70053

**Published:** 2025-05-01

**Authors:** Oumaima Marfouk, Ghalia Kaouane, Lara Leclerc, Fanny Munsch, Bei Zhang, Noël Pinaud, Rémy Gérard, Mickaël Fayon, Sophie Périnel‐Ragey, Eric Dumas de la Roque, Jérémie Pourchez, Yannick Crémillieux

**Affiliations:** ^1^ Institut des Sciences Moléculaires, CNRS, UMR 5255, Université de Bordeaux Bordeaux France; ^2^ Mines Saint‐Etienne, Université de Lyon, Université Jean Monnet, INSERM U1059 SAINBIOSE, Centre CIS Saint‐Etienne France; ^3^ Institute of BioImaging Université de Bordeaux Bordeaux France; ^4^ Canon Medical Systems Europe Zoetermeer The Netherlands; ^5^ Department of Pediatrics, Neonatal Intensive Care Unit Bordeaux University Hospital Bordeaux France; ^6^ Centre Hospitalier Universitaire de Bordeaux, Département de Pédiatrie, CIC‐P INSERM 1401 & Université de Bordeaux, Centre de Recherche Cardio‐thoracique de Bordeaux, INSERM U1045 Bordeaux France; ^7^ Université Jean Monnet, INSERM U1059 SAINBIOSE, Centre Hospitalier Universitaire de Saint Etienne Saint‐Étienne France

**Keywords:** automated segmentation, Gd‐based contrast agent, lung MRI, preclinical lung model, respiratory distress syndrome, surfactant, UTE

## Abstract

The administration of exogenous surfactant is essential for many premature infants to compensate for pulmonary immaturity and the absence of endogenous surfactant at birth. Exogenous surfactant delivery techniques are continually being refined to improve the management of these infants, with the goal of increasing therapeutic efficacy and decreasing the invasiveness of delivery protocols. Imaging is one of the tools available to achieve these goals. In this study, we established and applied an magnetic resonance imaging (MRI) protocol in a rabbit animal model to determine the intrapulmonary distribution of surfactant solution administered by the clinical reference method. The protocol was applied to an ex vivo model of isolated thorax from non‐valued food industry by‐products. The protocol made it possible to image surfactant biodistribution with isotropic spatial resolution in the millimeter range, to determine surfactant distribution between the main airways and distal lung regions where alveoli are present using automated segmentation techniques, and to quantitatively map the distribution of the administered surfactant solution.

## Graphical abstract

The administration of surfactant is essential to compensate for the lack of surfactant in the lung of very premature infants. MRI protocols can be used to visualize and quantify the surfactant distribution in lung to validate and optimize surfactant administration techniques for premature infants.

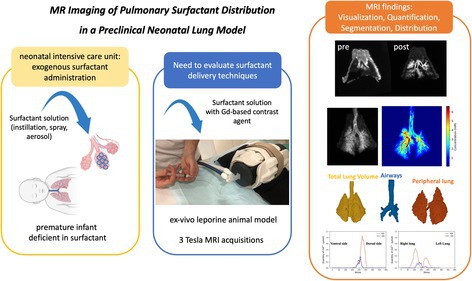

**Abbreviations**
CER: central enhanced region
IRDS: infant respiratory distress syndrome
LISA: less invasive surfactant administration
MIP: maximum intensity projection
PDF: peripheral distribution fraction
PER: peripheral enhanced region
PVF: peripheral volume fraction
TLV: total lung volume
UTE: ultra‐short echo time

## 1 Introduction

Infant respiratory distress syndrome (IRDS) and its consequences are a major cause of morbidity and mortality in preterm infants, accounting for approximately 15% of all neonatal deaths worldwide [1]. This life‐threatening respiratory condition, characterized by acute respiratory distress and the need for supplemental oxygen, is caused by a lack of surfactant due to lung immaturity. The administration of exogenous surfactant has been shown to improve outcomes and reduce mortality in preterm infants with IRDS and has become a cornerstone of IRDS therapy in neonatal intensive care units [2]. By improving lung function and preventing IRDS, the administration of surfactant can reduce the need for mechanical ventilation or shorten the duration of ventilatory support. This is crucial as mechanical ventilation can lead to complications such as lung injury, infection, and damage to the developing lungs of premature babies. The administration of surfactant can also help to prevent other lung diseases such as bronchopulmonary dysplasia and pneumothorax, which occur more frequently in premature babies with inadequate surfactant levels. Overall, the administration of surfactant to preterm infants helps to support their lung development, improves respiratory function, and reduces the risk of complications associated with preterm birth. European data from 2014 to 2016 show that around 50% of all babies born between 22 and 32 weeks received surfactant [3].

The techniques for administering exogenous surfactant have undergone several important developments since their first applications. Nowadays, the less invasive surfactant administration (LISA) technique is considered as the benchmark for surfactant delivery [4, 5]. This technique allows surfactant to be administered into the lungs of preterm infants without the need for intubation and mechanical ventilation and aims to reduce the invasiveness and potential risks associated with earlier surfactant administration methods. With the LISA technique, the infant is usually connected to a continuous positive airway pressure to support their breathing. A small catheter or feeding tube is carefully inserted into the trachea through the mouth or nose while the infant breathes spontaneously. The surfactant is then administered through the catheter in small aliquots.

Despite steady progress in exogenous surfactant delivery methods, there is still a need to improve delivery techniques, particularly in terms of safety, invasiveness, and efficacy, for example, by ensuring that as much surfactant as possible reaches the alveoli in order to effectively treat IRDS and improve lung function.

Due to the ethical, medical, and technical constraints for clinical investigations in a population of fragile preterm infants, the evaluation of exogenous surfactant administration can be performed in animal models such as preterm rabbits [6, 7] or young rabbits [8, 9], an animal model that closely resembles the weight and lung volume of preterm infants. In these preclinical studies, various imaging techniques (x‐ray and positron emission tomography) were used to directly or indirectly assess the distribution of the administered surfactant in the animal's lungs.

However, to our knowledge, the suitability of magnetic resonance imaging (MRI) to study the administration of exogenous surfactant has never been investigated. Despite the difficulty of using MRI in the lung, it has certain advantages. It allows three‐dimensional (3D) imaging with millimeter resolution and provides excellent contrast between different soft tissues. Importantly, no ionizing radiation is used, which is a major advantage for imaging very young children and the reason why lung MRI is being touted as a strong alternative to CT lung imaging in pediatrics and neonatology [10, 11]. The development of novel preclinical MRI methods to evaluate the treatment of IRDS can therefore be regarded as potentially transferable to clinical practice in premature and very young infants.

The ability to visualize the intrapulmonary distribution of aerosolized or instilled solutions by MRI has been demonstrated preclinically using MRI contrast agents added to administered solutions [12, 13]. In this study, we implemented and applied a similar approach to investigate the distribution of surfactant solution administered with the LISA technique in a rabbit lung model using MRI. Specifically, we sought to evaluate the potential of lung MRI to image surfactant concentration and to determine the distribution of surfactant between the airways and peripheral regions of the lung.

This proof‐of‐concept study was performed on isolated ex vivo leporine thoraces, ventilated by pleural‐mimicking depressions using a hypobaric chamber [14], to better assess the reproducibility of surfactant distribution measurements and to establish ventilation parameters and conditions such as continuous positive airway pressure, spontaneous breathing, and tidal volume that are as close as possible to those used in clinical practice. In the context of a feasibility study, the use of this ex vivo leporine model also enables compliance with the 3Rs approach by using unvalued by‐products from the food industry instead of live animals.

## 2 Methods

### 2.1 Preparation of Leporine Lungs and Passive Ventilation

Rabbits (Hyplus breed) were obtained from slaughterhouse (Fougerouse, Saint‐Anthème, France), satisfying French sanitary controls. After slaughter, skinned rabbits were around 1.8 kg of body weight. The thorax was isolated by dissection, and visual control of lung, trachea, and pleura integrity was performed before freezing them at −20°C.

**FIGURE 1:**
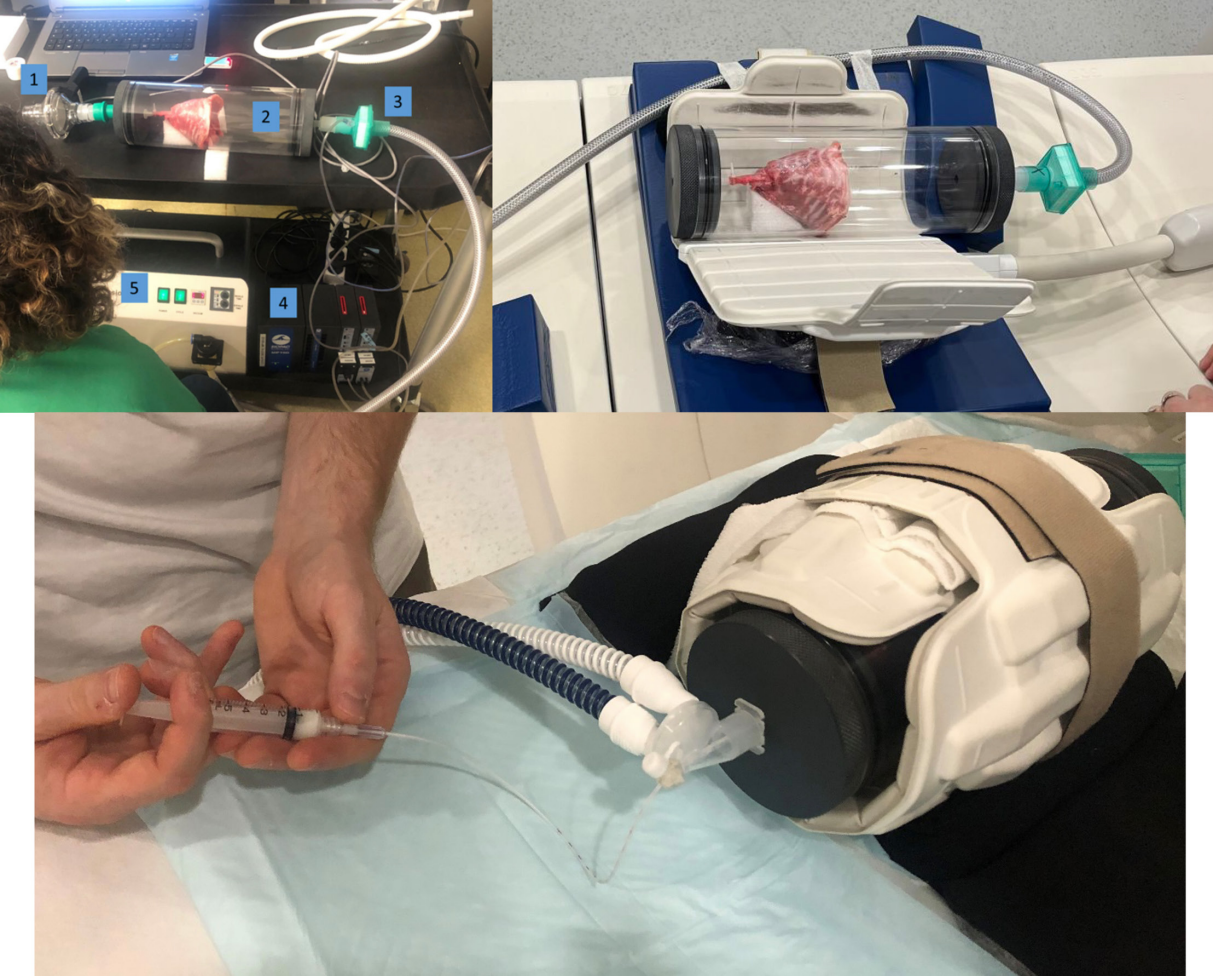
Top left image: Overview of the experimental set‐up for lung function measurements. The pneumotachograph (1), used for recording the airflows and connected to the trachea. At the other end of the hypobaric chamber (2), a pressure line with a filter (3) connected to the negative pressure generator (5). The BIOPAC system (4) used to drive the application of negative pressure and to record the lung volume. Top right image: Hypobaric enclosure containing an isolated ex vivo rabbit thorax in the unwrapped 16‐channel flexible MRI coil. Bottom image: Instillation of the surfactant solution into the lungs with the MRI flexible coil wrapped around the hypobaric chamber.

Thoraces were thawed a few hours before the experiments and were placed supine in a cylindrical and sealed chamber of 100 mm in diameter and 300 mm long. On one side of the chamber, the lung trachea was airtightly attached to a connecting tubing used for lung function measurements, application of continuous positive airway pressure, and administration of the surfactant solution. On the other side of the chamber, connection was established with a depression generator SuperDimension (Covidien, Dusseldorf, Germany) used to mimic pleural depression. Recruitment of each lung was carried out, and lungs were ventilated similarly to in vivo passive ventilation. A neonatal ventilator (Babylog, Draeger, Lübeck, Germany) was connected to the trachea to maintain a continuous positive airway pressure fixed at 6 cmH_2_O. A heated humidifier was also connected on ventilator circuit. Six leporine thoraces were used for the experiments. A photograph of the experimental setup for lung function measurements is shown in Figure 1.

### 2.2 Lung Function Measurements

Before surfactant instillation, lung function measurements were performed, as described previously in details [14], with a BIOPAC system (Biopac, Goleta, USA) composed of a pneumotachograph (TSD117) and a differential pressure transducer (TSD160D), which were connected to amplifier (DA100C) plugged on unit acquisition (M160). This system allowed a real‐time follow‐up of depression in the enclosure and airflow at the trachea. AcqKnowledge 5.0 software was used to set and measure respiratory parameters for each ventilation cycle: tidal volume, respiratory rate, inspiratory time, expiratory time, and compliance.

Data were recorded for at least five cycles with fixed inspiratory and expiratory time of 0.18 and 1.11 s, respectively, corresponding to a total breathing cycle of 1.29 s or 47 breathes per minute. Breathing patterns and ventilation parameters were chosen to represent as closely as possible those of preterm infants in intensive care unit. The same breathing patterns and ventilation parameters were applied on the lungs during the MRI acquisitions.

During the MRI scans, the lungs were ventilated by applying a cyclic negative pressure in the hypobaric chamber. A positive pressure of 6 cmH_2_O was applied at the level of the trachea to simulate the clinical conditions of surfactant administration using the LISA method, in which a continuous positive airway pressure is applied.

### 2.3 Preparation and Administration of the Instilled Solution

The solution instilled into the animal's lung was prepared by mixing 2.7‐mL solution of a clinically used surfactant (Curosurf, Chiesi Farmaceutici, Parma, Italy) and 0.27 mL of a clinically used Gd‐based contrast agent (Dotarem, Guerbet, Villepinte, France). The solution to be instilled was mixed and heated in a water bath at 37°C for 15 min. 2.5 mL of the solution at body temperature was then filled into a syringe connected to a catheter of 40 cm long, 0.8 mm inner diameter, 1.5 mm outer diameter, and 0.3 mL dead volume (Vygon, Ecouen, France). Given the average weight of the animals and the 2.2 mL volume of solution effectively instilled into the lungs, the amount of surfactant administered was approximately 100 mg/kg body weight. Given the initial concentration of 500 mM Gd^3+^ in the contrast solution, the 2.2 mL of solution instilled corresponded to a total of approximately 100 μmol of Gd^3+^ delivered to the lungs.

The catheter was then inserted, through the connection of the neonatal ventilator, into the trachea of the lung in the hypobaric chamber. The catheter was inserted into the trachea until its tip was about 3 cm above the carina. A piece of modelling clay was used at the entry point of the catheter to ensure an airtight fit and maintain the continuous positive airway pressure at 6 cmH_2_O. The solution was then instilled into the lungs (Figure 1) in approximately 5 s, and the patient bed was slid into the scanner to position the coil with the housing in the center of the magnet for MRI imaging.

### 2.4 MRI Acquisitions

MRI acquisitions were performed on a 3‐T whole‐body magnet (Vantage Galan 3T ZGO, Canon Medical Systems Corporation, Japan) 7 min after the surfactant instillation. The nonmagnetic hypobaric chamber containing the isolated ex vivo rabbit thorax was positioned in a 16‐channel Flex medium coil (Canon Medical Systems Corporation, Japan) wrapped around the chamber (Figure 1) and centered in the MRI scanner. Imaging was performed with the whole‐body coil for RF excitation and the flexible MRI coil for reception.

MR lung images were acquired using a 3D ultra‐short echo time (UTE) MRI sequence with the following acquisition parameters, repetition time = 3.7 ms, echo time = 96 μs, 1 average, total acquisition time = 3min 28 s, field of view = 11.3 × 11.3 cm^2^, slice thickness = 1 mm, voxel size = 0.78 × 0.78 × 1 mm^3^. The data were recorded in a radial pattern, with the starting point of the radial lines in the center of k‐space and the end points distributed over the surface of a sphere. Gradient delay correction was applied, and images were generated online using the manufacturer reconstruction software installed on the MRI scanner. The 3D UTE images were acquired with four different excitation flip angles α = 5°, 8°, 16°, and 25° to obtain different magnitudes of T_1_ weighting in the lung images. The 3D UTE images were acquired twice, before and after instillation of the 2.2‐mL surfactant solution. The total acquisition time required for the flip angle adjustment and shimming procedures, the acquisition of localizer images, and the eight 3D UTE datasets took approximately 40 min per animal thorax.

During the MRI scans, the lungs were ventilated as described earlier by applying a cyclic negative pressure in the hypobaric chamber. A positive pressure of 6 cmH_2_O was applied to the respiratory tract to simulate the clinical conditions of surfactant administration using the LISA method with a continuous positive airway pressure.

### 2.5 Quantitative Imaging of the Contrast Agent Distribution

The signal enhancement SE was defined as (SNR_post_ − SNR_pre_)/SNR_pre_ where SNR_post_ and SNR_pre_ are the signal‐to‐noise ratio (SNR) measured, respectively, in regions of the lungs before and after administration of the surfactant solution with contrast agent.

The concentration C (mM) of the contrast agent in the lung was calculated using the relationship

(1) $$C=\frac{1}{r_{1}}\left(R_{1\text{post}}-R_{1\mathrm{pre}}\right),$$

where *r*_1_ is the longitudinal relaxivity of the contrast agent induced by the Gd^3+^ ion and *R*_1pre_ and *R*_1post_ are the longitudinal relaxation rates in the lung tissue before and after administration of the surfactant solution.

Longitudinal relaxation rates *R*_1_ = 1/T_1_ were obtained using the known analytical expression of the MRI signal amplitude S for the UTE imaging sequence [15].

(2) S=A.sinα1−e−TRT11−cosα.e−TRT1,

where *A* is a constant depending on the MRI instrumentation and on the nuclear magnetization at thermal equilibrium, α the flip angle, *TR* the repetition time, *TE* the echo time, and *T*_1_ the longitudinal relaxation time.

*R*_1pre_ was obtained by measuring, in the UTE images acquired at four different flip angles, the MRI signal intensity in a region of interest corresponding to a distal region of the lungs, excluding large airways. The *R*_1post_ values were computed pixel‐by‐pixel by measuring the signal intensity value in the UTE image acquired with a 25° flip angle. The value of the *A* constant was obtained by measuring the image signal intensity in a region of interest located in a distal region of the lungs free of solution with contrast agent (absence of signal enhancement). The concentration of the contrast agent was then computed pixel‐wise using Equation (1) with the *r*_1_ value of the contrast agent set at 3 mM^−1^ s^−1^ per Gd^3+^ ion as reported for human plasma at 3 T [16].

### 2.6 MRI‐Based Lung Segmentation

The MR images of the lung were segmented into three regions, referred to as central enhanced region (CER), peripheral enhanced region (PER), and total lung volume (TLV). The CER corresponds to the intrapulmonary areas, identified as central airways on the MR images, with MR signal hyperintensities due to the presence of the Gd‐based contrast agent solution. The PER corresponds to the distal airways and intrapulmonary areas, with the exclusion of the CER, with MR signal hyperintensities due to the presence of the Gd‐based contrast agent solution. The TLV corresponds to the total volume identified as lung tissue on the MR images (including the airways) regardless of the presence or absence of surfactant and contrast agent solution.

**FIGURE 2:**
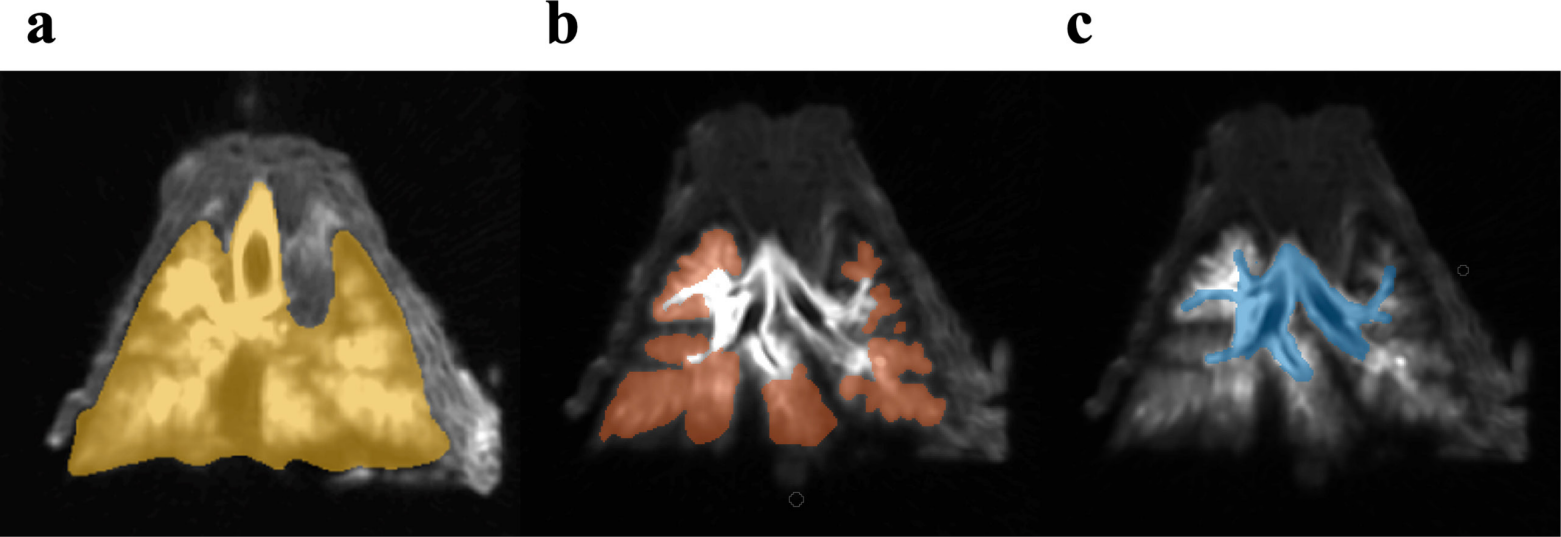
Example of the three delineated lung regions using manual segmentation in a coronal slice: (a) total lung volume TLV, (b) peripheral enhanced region PER, and (c) central enhanced region CER.

Volume Segmenter, a MATLAB (The MathWorks, Natick, MA, USA) tool for manual segmentation of volumetric image data, was used for manual segmentation of the three lung regions. Five 3D UTE datasets, acquired with a flip angle of 25°, were manually segmented to identify the PER and TLV regions. These segmentations were later used to train deep learning models as detailed below. CER was determined as a complement to PER to capture the totality of lung regions enhanced by the presence of surfactant and contrast agent solution. Examples of delineations using manual segmentation of these three regions are shown in Figure 2.

**FIGURE 3:**
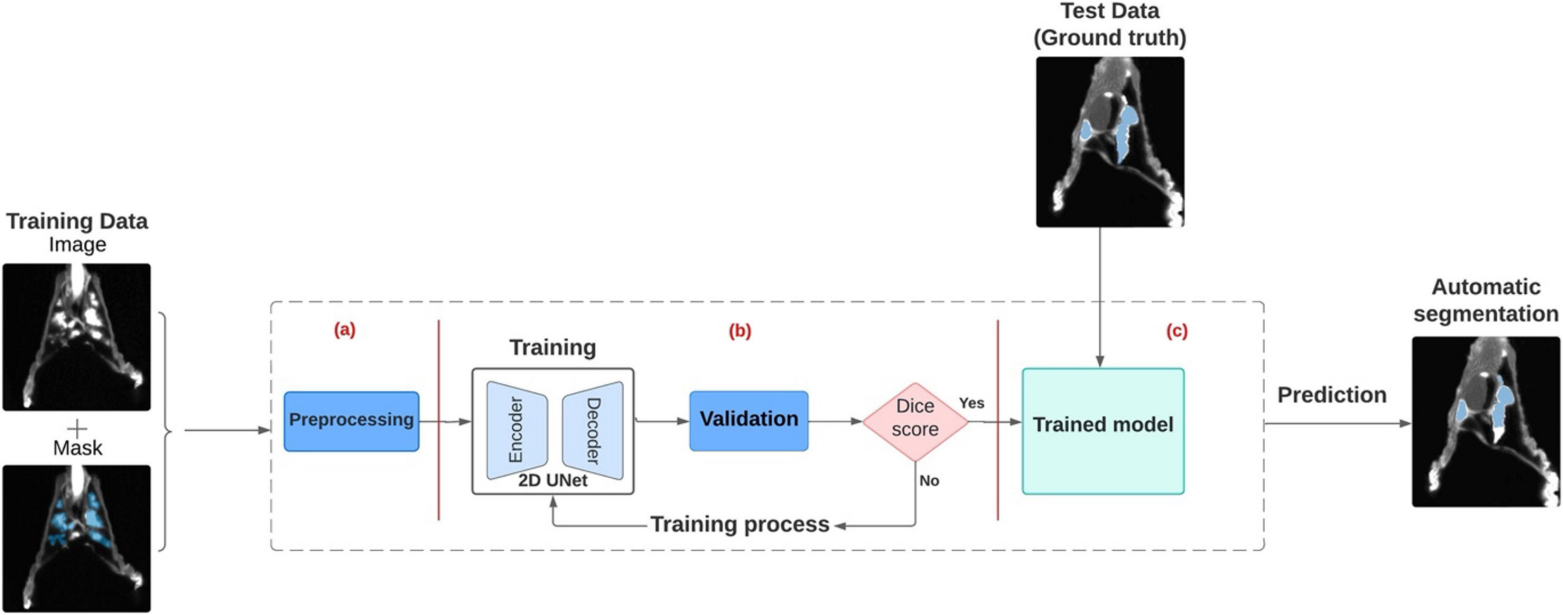
Automatic segmentation pipeline. (a) The preprocessing of the data, which may involve the use of a filter to remove noise or to enhance contrast, normalized intensity or magnify data. (b) The training process consisted of an iterative trial and error in selecting a set of hyperparameters and architectural configurations, and the performance of the model was monitored against a validation set. (c) Once the Dice score–based evaluation of segmentation quality was completed, the model was used on a test dataset.

The nnU‐Net deep learning framework [17] was used to automatically segment the TLV and PER. The nnU‐Net framework introduced a data‐driven heuristic, the selection of hyperparameters, which proved to be very efficient for small datasets. The training scheme described in Figure 3 was used to train a peripheral zone segmentation model. The automatic segmentation network was trained over 1000 epochs with a batch size of 32 to ensure robust model learning and performance optimization. The dataset consisted of 3D MRI images in DICOM format and a 3D segmentation in mat format. To ensure uniformity and compatibility with the training models, all data were converted to the widely used NIFTI format. Python scripts were developed for data analysis and visualization and executed in the Anaconda distribution environment using Spyder's integrated development environment. MATLAB Volume Viewer was then used to visualize the segmented anatomical structures, explore their spatial relationships in a 3D context, and generate 3D maximum intensity projections (MIPs) from the MR images.

### 2.7 MRI Metrics

Two MRI metrics were defined to assess the distribution of surfactant solution in the lung.

The peripheral distribution fraction (PDF) was defined as the quantity of Gd^3+^ ions in the PER voxels over the total quantity of Gd^3+^ ions in the PER and CER voxels, $\mathrm{DF}=\frac{\sum_{\text{voxel}}^{\mathrm{PER}}Q_{\mathrm{Gd}}}{\sum_{\text{voxel}}^{\mathrm{PER}}Q_{\mathrm{Gd}}+\sum_{\text{voxel}}^{\mathrm{CER}}Q_{\mathrm{Gd}}}$, where Q_Gd_ corresponds to the quantity of Gd^3+^ in each voxel. Because the voxel volumes were identical, the value of the Gd^3+^ concentration obtained in each voxel was used to calculate the PDF value.

The PDF value, expressed in %, was chosen as an indicator for the partitioning of the surfactant solution between the airways and the peripheral lung regions.

The peripheral volume fraction (PVF) was defined as the proportion of the volume corresponding to the PER over the total volume of the lung, $\mathrm{PVF}=\frac{\sum_{\mathrm{PER}}\mathrm{vol}}{\sum_{\mathrm{TLV}}\mathrm{vol}}$, where *vol* corresponds to the volume of a voxel.

The PVF value, expressed in %, was chosen as an indicator of the proportion of the peripheral regions of the lung reached by the surfactant solution.

### 2.8 In Vivo Experiment

To establish a point of comparison with the ex vivo acquisitions, an experiment was performed on a 6‐week‐old female White New Zealand rabbit of 1.1 kg body weight (Charles River, Saint‐Germain‐Nuelles, France). After a pre‐sedation with medetomidine (0.35 mg/kg), the animal was anesthetized with a solution of ketamine (50 mg/kg) and xylazine (7 mg/kg). A catheter (Surfcath, Vygon, France) dedicated to the administration of surfactant to premature/neonates using the LISA method was then inserted beyond the animal's vocal cords. 2.2 mL of surfactant and contrast agent solution identical to the one used in ex vivo experiments were slowly instilled at 0.25 mL/s in the lungs of the animal lying supine. The animal was then positioned supine inside the MRI scanner, and the MRI acquisitions were performed 7 min after the administration of the surfactant. MRI acquisition was also performed before the administration of the surfactant solution. The MRI coil and sequence parameters used were identical to those used in ex vivo experiments.

This in vivo protocol was approved by the local animal welfare committee (University of Bordeaux, reference number APAFIS no. 49689‐2024052813567785), and the animal experimental procedure was conducted in compliance with EU guidelines (directive 2010/63/EU) and the ARRIVE recommendations.

## 3 Results

### 3.1 Lung Functional Measurements

For the six thoraces used in this study, the mean delta P was measured at −88 ± 2.1 cmH_2_O, and mean compliance was calculated at 0.133 ± 0.005 mL/cmH_2_O. These values were obtained with the ventilation parameters set at 12 mL for tidal volume with 47 cycles/min. Mean inspiratory time was 0.181 s, and mean expiratory time was 1.110 s. The individual measurements are summarized in Table 1.

**TABLE 1:** Respiratory parameters, delta P and compliance, measured for each thorax before administration of the surfactant.

Thorax	Delta P (cmH_2_O)	Compliance (mL/cmH_2_O)
Specimen #1	−97 ± 0.0	0.138 ± 0.006
Specimen #2	−94 ± 0.8	0.134 ± 0.006
Specimen #3	−79 ± 5.0	0.134 ± 0.004
Specimen #4	−110 ± 1.0	0.098 ± 0.005
Specimen #5	−62 ± 0.4	0.165 ± 0.007
Specimen #6	−89 ± 5.4	0.131 ± 0.003
Mean ± SD	−88 ± 2.1	0.133 ± 0.005

### 3.2 Contrast‐Enhanced MRI of Surfactant Solution

**FIGURE 4:**
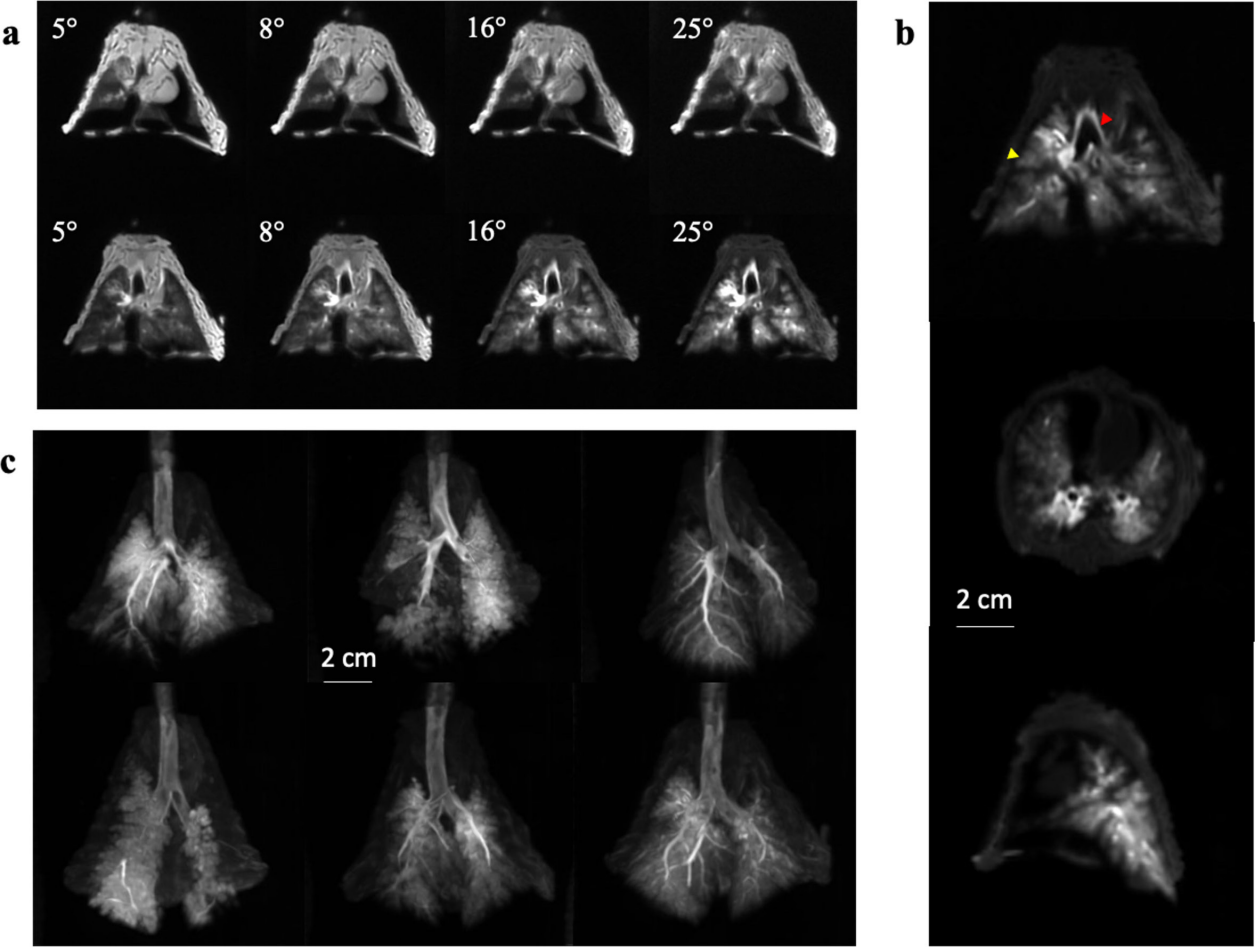
(a) Coronal MRI slices, acquired before (top row) and after (bottom row) administration of the surfactant and contrast agent solution, at the four excitation flip angles. (b) From top to bottom: coronal, axial, and sagittal slices corresponding to the isolated lungs in (a) acquired at a flip angle of 25°. The red arrowhead and the yellow arrowhead indicate, respectively, the inner enhancement of the left bronchi and one of the enhanced acini in the right lung. (c) MIP reconstruction of the six isolated rabbit lungs imaged after administration of the solution. The lungs are seen from the front of the thorax. These images were acquired approximately 25 min after the surfactant administration.

Examples of lung MRI images as a function of the flip angle are shown in Figure 4a. The 1‐mm‐thick coronal slices in the top row were acquired before administration of the surfactant solution. Due to its low tissue density, the lung parenchyma appeared hypointense on the MRI images compared to the chest muscles, trachea, and main bronchi. Variations in flip angle resulted in little change in image contrast, indicating similar T_1_ values in all tissues.

The images in the bottom row were acquired after administration of the 2.2‐mL surfactant solution with added MRI contrast agent. Enhancement of the airways and lung parenchyma where the solution was distributed was visible at all flip angles, indicating shortened T_1_ values due to the contrast agent.

For this isolated thorax, the signal enhancement SE measured in the peripheral contrast‐enhanced regions of the lung was found to be 400%, 620%, 1270%, and 1940% for flip angles of 5°, 8°, 16°, and 25°, respectively. The images acquired with the 25° flip angle, which showed the highest image contrast and the highest enhancement in the regions with surfactant solution, were used for subsequent qualitative and quantitative image analysis.

Figure 4b shows typical coronal, axial and sagittal slices acquired with a 25° flip angle. The spatial resolution of the images (0.78 × 0.78 × 1 mm) allowed the visualization of airways with a diameter of a few millimeters, which corresponds to the fifth‐ or sixth‐generation airways in rabbits [18]. The lumens of the main airways were also visible up to the third generation. Signal enhancement on the inner walls of these main airways, corresponding to the presence of the administered solution, was observed over a thickness of less than 2 mm (red arrowhead). Acinar structures several millimeters in size were also seen in the distal part of the lungs as shown in Figure 4b (yellow arrowhead).

MIP reconstructions are shown in Figure 4c for each of the rabbit lungs imaged after administration of the surfactant solution with MRI contrast agent. These images qualitatively illustrate the differences in the distribution of surfactant solution from one isolated lung to another. These disparities were observed in the distribution of the solution between the right and left lung, in the homogeneity of the solution distribution in the peripheral zones of the lung and in the partitioning of the solution between the main airways and the distal parts of the lung.

### 3.3 Quantitative Imaging of Surfactant Solution

**FIGURE 5:**
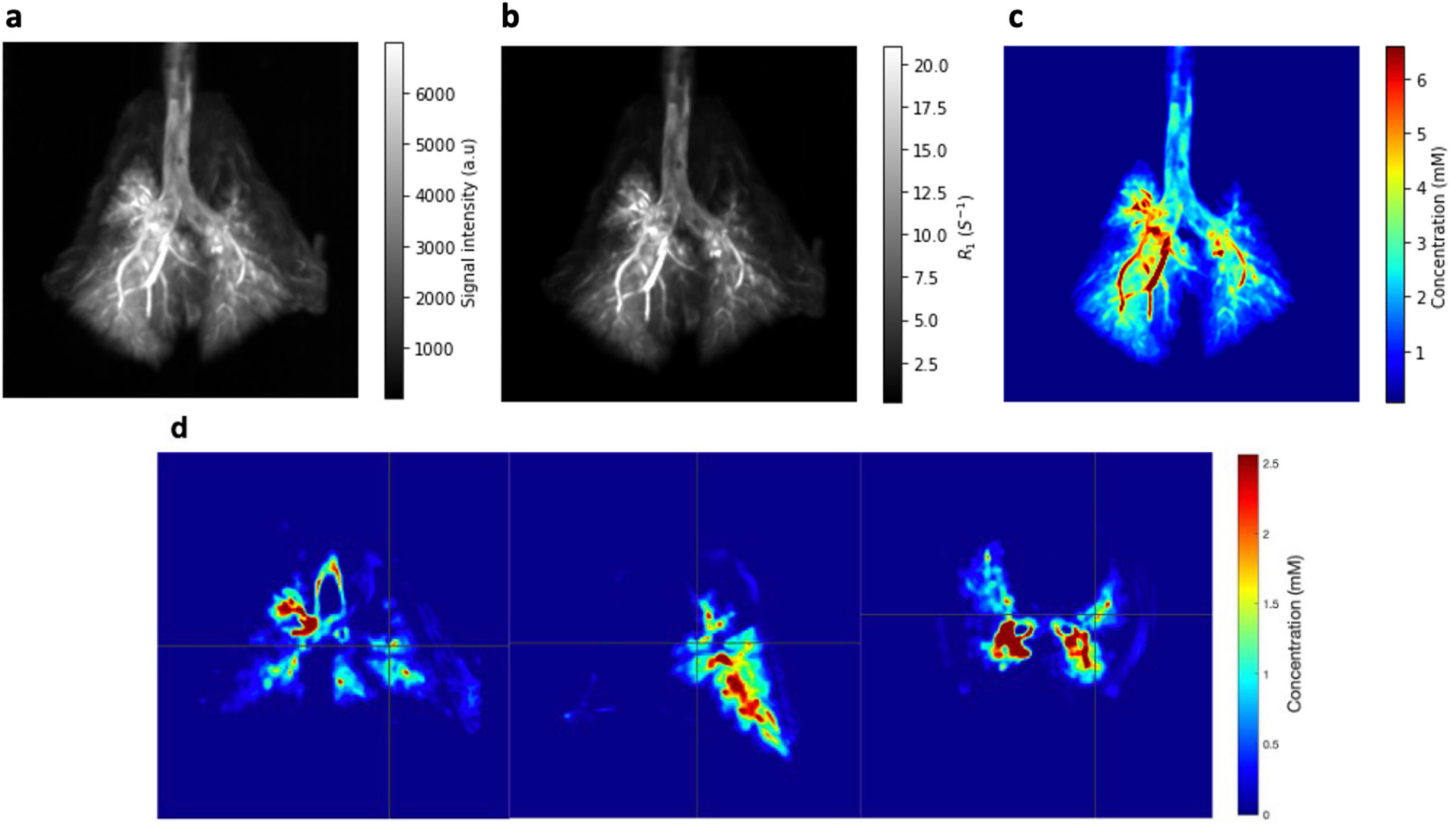
(a) MIP reconstruction of a rabbit thorax imaged after administration of the surfactant solution, (b) corresponding MIP reconstruction of the R_1_ map, and (c) corresponding MIP reconstruction of the contrast agent concentration in the instilled surfactant solution. The MIPs are displayed as seen from the front of the thorax. (d) From left to right: Examples of coronal, sagittal, and axial 1‐mm‐thick slices of contrast agent concentration map extracted from the 3D dataset.

MIPs of longitudinal relaxation rate R_1_ and Gd^3+^ concentration in Figure 5 illustrate quantitative MRI of the surfactant distribution in the lungs. The mean T_1_ values post‐administration and the computed Gd^3+^ concentration in the contrast‐enhanced lung regions are reported in Table 2. The average T_1_ value after surfactant administration was 266.2 ± 88.9 ms, and the mean value of Gd^3+^ concentration was 2.6 ± 1.6 mM. Table 2 includes as well the contrast‐enhanced volume for each specimen and the corresponding amount of Gd^3+^ ion measured in each lung volume.

**TABLE 2:** Contrast‐enhanced lung volumes for each specimen and the corresponding mean T_1_ value post‐injection, Gd^3+^ concentration, and quantity of Gd^3+^ ion measured in these lung regions.

Thorax	Contrast‐enhanced lung volume (mL)	T_1_ post‐administration (ms)	Gd^3+^ concentration (mM)	Amount of Gd^3+^ (μmol)
Specimen #1	31.8	170	4.6	146.3
Specimen #2	39.1	285.3	1.8	70.4
Specimen #3	45.8	318.9	1.4	64.1
Specimen #4	30.9	166.5	4.6	142.1
Specimen #5	37.7	259.7	2.5	94.3
Specimen #6	48.9	397	0.9	44
Mean ± SD	39 ± 7.3	266.2 ± 88.9	2.6 ± 1.6	93.5 ± 42.4

**FIGURE 6:**
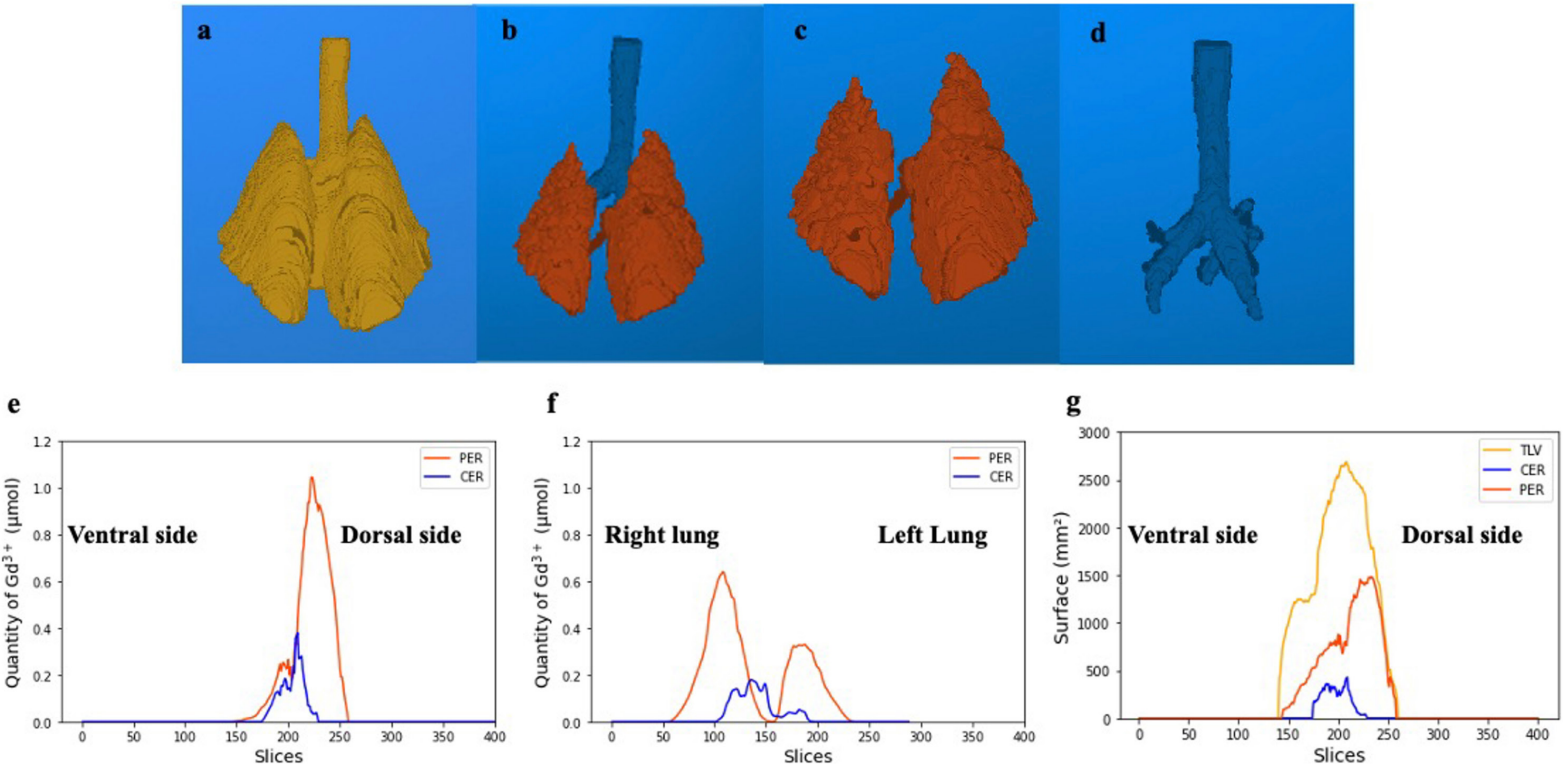
3D volume rendering views of (a) the TLV, (b) the PER in red and CER regions in blue, (c) the PER alone, and (d) the CER alone. The three regions were obtained by automatic segmentation. The isolated lungs are viewed from the back. (e) Ventral‐to‐dorsal distribution of the quantity of Gd^3+^ in the segmented regions CER and PER. (f) Right‐to‐left lung distribution of the quantity of Gd^3+^ in the segmented regions CER and PER. (g) Ventral‐to‐dorsal distribution of the surface of the segmented CER, PER, and TLV regions.

Examples of the automatic nnU‐Net segmentation of the lungs used to provide regional quantitative information on surfactant distribution are shown in Figure 6. Figure 6a displays the segmentation of the total lung volume TLV, corresponding to all the lung regions, regardless of MRI signal enhancement induced by the solution. Figure 6b shows the segmentation of the peripheral contrast‐enhanced and central contrast‐enhanced regions obtained from the same isolated lungs. These two segmented regions are displayed separately in Figure 6c and Figure 6d. These 3D volume visualizations are dorsal views of isolated thoraces. Four generations of airways were identified in the segmented CER 3D dataset. The mean volumes and standard deviations measured on all thoraces were 91 ± 5.4 mL, 34.3 ± 6.8 mL, and 4.75 ± 0.9 mL for the TLV, PER, and CER, respectively.

Figure 6e–g illustrate regional measurements obtained from the same segmented dataset. Figures 6e and 6f show, respectively, the distribution of the quantity of Gd^3+^, derived from slice‐by‐slice measurements, from ventral to posterior and from left to right regions of the lungs. The ventral‐to‐dorsal distributions of the surface area, expressed in mm^2^, of the segmented total lung, central airways and peripheral lung regions measured for each coronal slice are shown in Figure 6g. The equivalent plots obtained for each of the six imaged lungs analyzed are shown in the Supporting Information.

As expected from the supine position of the thorax during instillation of the solution and acquisition of the MRI images, the ventral‐to‐dorsal distributions in the peripheral lung regions highlighted the preferential distribution of surfactant solution in the dependent regions of the lungs. In four out of six isolated lungs, the right/left distribution indicates a greater distribution of solution in the peripheral lung regions of the right lung. This asymmetry in distribution between the right and left lungs probably reflects the asymmetry in right and left lung volumes and the geometry of the tracheal bifurcation into the right and left main bronchi.

The values of the quantitative metrics PDF and PVF for each isolated thorax are shown in Table 3. The mean value of the surfactant fraction observed in the peripheral region of the lungs was 84.02% ± 5.24%, reflecting a large and relatively constant proportion of surfactant in the peripheral regions of the lungs. The proportion of volume occupied by surfactant to the total lung volume, which was 32.51% ± 5.28%, also showed minimal variations from one isolated thorax to another.

**TABLE 3:** Summary of PDF and PVF values for each specimen of isolated thorax.

Thorax	Peripheral distribution fraction PDF (%)	Peripheral volume fraction PVF (%)
Specimen #1	83.0	35.7
Specimen #2	87.4	38.8
Specimen #3	79.1	23.6
Specimen #4	92.1	29.4
Specimen #5	78.1	33.7
Specimen #6	84.6	33.7
Mean ± SD	84.0 ± 5.2	32.5 ± 5.3

### 3.4 In Vivo MRI Acquisition

**FIGURE 7:**
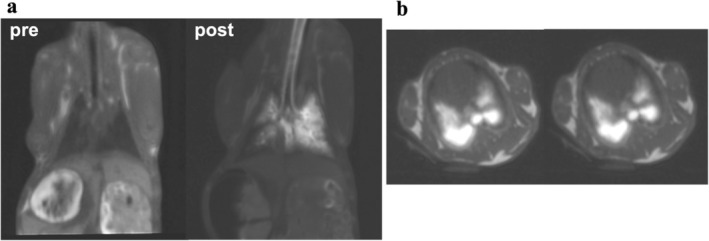
(a) Coronal slices acquired in vivo before and after the administration of the surfactant. (b) Axial slices acquired in vivo after the administration of the surfactant. The two images were acquired 30 min apart.

Coronal slices acquired before and after surfactant administration are shown in Figure 7a. The signal enhancement measured in the peripheral contrast‐enhanced regions of the lung was found to be 1700% for an excitation flip angle of 25°, corresponding to the maximum signal enhancement.

As shown in the axial slices in Figure 7b, the distribution and signal intensity of the surfactant solution remained unchanged for 30 min, and no signal enhancement was observed in other tissues, indicating that there was no passage of the contrast agent from the airspace to the vascular space and that the colocalization of the contrast agent and surfactant was maintained during this period. Some surfactant solution, presumably expelled from the airways, was observed in the esophagus of the animals.

## 4 Discussion

The aim of the lung ventilation measurements carried out on isolated thoraces, prior to MRI scans, was to ensure lung integrity (absence of leakage and homogeneous inflation of both lungs) and their ability to ventilate. These measurements were carried out at tidal volumes and respiratory rates as close as possible to those found in spontaneously breathing infants. Delta P and compliance values measured with these ventilatory parameters showed very good reproducibility between the different specimens, indicating similar lung harvesting and preservation conditions.

The UTE MRI sequence was used to image the distribution of surfactant in the lungs. This MRI sequence is recognized as the most appropriate one for visualizing lung tissue and structures with the highest spatial resolution and a good contrast between tissues. Its UTE avoids rapid MRI signal decay in the lungs and limits artifacts due to cardiac and, in this study, to pulmonary motion. This sequence has been applied in adults and young children, including infants, to study pathologies such as fibrosis, cystic fibrosis, or bronchopulmonary dysplasia [11, 19, 20]. Its use has also been described in preclinical animal models ranging from mice to pigs.

This sequence was particularly appropriate for this study to avoid motion artifacts associated with ventilation of the lungs during image acquisition and to provide millimetric spatial resolution for visualization of airways and lung structures in this small‐size leporine lung model. The sequence also provided T_1_‐weighted images that are well suited for the use of positive gadolinium‐based contrast agents. The administration of this type of contrast agent via the airways has been previously demonstrated in isolated lungs [12], animal models [21, 22], and humans [23]. The gadolinium‐based contrast agent was necessary in this study to highlight the presence and distribution of surfactant solution in the lungs. This approach for imaging the surfactant distribution requires that the colocalization of contrast agent and surfactant is maintained within the lung during image acquisition. This assumption is supported by the absence of blood perfusion, a source of contrast agent clearance from the lung in this isolated lung model, and the lack of change in successive images acquired over 20 min (data not shown). In order to draw relevant conclusions from the distribution images obtained, it is also important that the contrast agent solution added to the surfactant does not cause excessive changes in the properties of the surfactant solution, such as its viscosity, which could affect its distribution in the lungs. In this study, the addition of the contrast agent solution was limited to 10% by volume, with no visible effect on the viscosity of the mixture. Of note, the MRI signal enhancement measured in the study is significantly large, on the order of 1900%, and it is conceivable that the amount of contrast agent added to the surfactant solution could be halved, if necessary, without affecting the ability to image the distribution of the surfactant solution.

The relaxivity of the contrast agent was used to obtain a quantitative assessment of the intrapulmonary concentration of the contrast agent and, by extension, of the surfactant. This measure of contrast agent concentration was obtained using the known relaxivity of the contrast agent in human plasma. It is interesting to note that using this relaxivity value, the average amount of Gd^3+^ measured in the lungs, 93.5 μmol, was very close to the amount of Gd^3+^ actually instilled into the lungs, which was 100 μmol. Although the mean value of Gd^3+^ amount obtained for all isolated thoraces was close to the expected amount of delivered Gd^3+^, there were significant variations in the Gd^3+^ amount measured from one thorax to another. These variations were probably due in part to experimental fluctuations in the concentration and effective volume of solution administered, but more likely to the combined effect of imprecisions in the measurement of relaxation rates before and after solution administration and in the volume of contrast‐enhanced regions of the lung. Nevertheless, regardless of the imprecision in the absolute value of the concentrations measured in the lung, a 3D map of the relative concentration of Gd^3+^ can be generated. This allows the signal intensity in the T_1_‐weighted UTE images, depending on the parameters of the sequence used, to be converted into quantitative information about the surfactant distribution in the lung.

The segmentation of the 3D lung images to discriminate the presence of surfactant in peripheral lung regions or in the central airways is particularly challenging due to the anatomical complexity of the lung and the subtle intensity variations in the images. Manual segmentation is possible but is time consuming, requiring several hours per dataset for a trained operator. The automated segmentation approach based on deep learning techniques implemented in the study, which typically took between 3 and 10 min, proved to be robust, accurate, and fast for the delineation of the PER and CER of the lung and for the total lung volume. This segmentation of the PER and CER was essential for assessing the effectiveness of surfactant distribution in the lung. Indeed, the presence of surfactant in the peripheral regions, particularly within the alveoli, is required to reduce alveolar surface tension and ultimately improve lung function. In this study, the coefficients of variation for the segmented volumes TLV, PER, and CER were 5.9%, 19.8%, and 18.9%, respectively, indicating a total lung volume very similar to that expected for animals of standardized weight and a similar variability for the volume occupied by surfactant solution in the peripheral lung and central airways.

After mapping the contrast agent concentration and segmenting the lung into three regions, the inter‐ and intra‐regional distributions of surfactant were determined. In this study, the fraction of surfactant in the peripheral region of the lung PDF was found to be very similar from one isolated thorax to another, with a coefficient of variation of 6.2%. Similarly, the fraction of total volume PVF occupied by the surfactant solution varied moderately from one isolated thorax to another, with a coefficient of variation of 16.2%. In addition, the distribution of surfactant concentration within the peripheral lung region revealed marked asymmetries in surfactant distribution along the anterior–posterior direction and between the left and right lungs.

This study was designed and performed with the aim of using the visualization and quantification of surfactant distribution to optimize and validate new approaches to surfactant administration in premature infants. In this feasibility study, the use of isolated ex vivo thoraces obtained from non‐valued by‐products of the food industry was motivated by both ethical and experimental considerations, given the possibility of easily controlling and modifying experimental ventilation parameters such as volume, positive pressure, lung positioning, and surfactant administration protocols.

The transfer and the validation of this approach in a second stage to in vivo models remains necessary to assess for example the efficacy of modified surfactant administration protocols in reducing respiratory distress syndrome. Under in vivo conditions, in the presence of lung perfusion, it will be necessary to ensure that colocalization of surfactant and contrast agent is maintained during MRI acquisitions. Previous results in murine animal models [21] have shown that the elimination half‐life of this type of contrast agent in the lung is in order of half an hour, which is compatible with acquisition times of a few minutes required to obtain lung images. In this regard, preliminary results obtained in vivo using the same surfactant delivery protocol indicate that the surfactant and contrast agent solution remain confined in the alveolar space for a period of time compatible with the time required for the MRI protocol. Furthermore, the in vivo distribution of the surfactant solution was very similar to that obtained in the ex vivo model. It should be noted that the spatial resolution in the ex vivo MRI acquisitions is higher than that observed in vivo, probably due to the absence of cardiac motion. Taken together, these observations are a good indicator of the relevance of the ex vivo model as a first step in evaluating and improving surfactant delivery to the lung.

The methodology used in this study has certain limitations that should be mentioned. First, the intrapulmonary administration of MRI contrast agents is currently not applicable to premature infants for safety reasons. Additionally, MRI of premature infants, especially those requiring surfactant administration, is extremely difficult to conceive in clinical practice. Another limitation of the model is the use of isolated thoraxes, which, according to our measurements, have a low compliance, 0.133 mL/cmH_2_O on average, due to postmortem rigidity. These low values represent a possible limitation of the ex vivo model for the extrapolation of the results to a healthy in vivo animal model. On the other hand, it should be noted that these compliance values are close to those measured in premature infants with IRDS, which are on the order of 0.43 mL/cmH_2_O/kg [24]. The same applies to the use of IRDS animal models where low pulmonary compliance is likely to be encountered.

Overall, the results presented in this study show that it is possible to obtain relevant quantitative information to assess surfactant distribution with millimeter spatial resolution in an ex vivo animal model. As a proof of concept, the study focused on the LISA method, considered as the current clinical reference for surfactant administration. However, the methodology reported here can in principle be applied to other surfactant delivery modalities such as aerosol, nebulization, and spray. Beyond its application to surfactant administration, this ex vivo animal model and the MRI approach reported here are applicable to other inhalation‐based drug therapies in order to assess drug distribution in the lungs.

## Supporting information


**Figure S1** Distribution of the chelated Gd^3+^ quantity (μmol) in the central enhanced regions (CER) and peripheral enhanced regions (PER) of the lungs for the six specimens of isolated thoraxes. Left plots: Ventral‐to‐dorsal distribution. Right plots: Right‐to‐left distribution.

## Data Availability

The data that support the findings of this study are available from the corresponding author upon reasonable request.
