# Supplementary material for: MR Imaging of Pulmonary Surfactant Distribution in a Preclinical Neonatal Lung Model
Source: NMR Biomed. 2025 May 1;38(6):e70053. doi: 10.1002/nbm.70053 (PMC12045669; doi:10.1002/nbm.70053)
Supplement: Supplementary file 1 — Figure S1 Distribution of the chelated Gd3+ quantity (μmol) in the central enhanced regions (CER) and peripheral enhanced regions (PER) of the lungs for the six specimens of isolated thoraxes. Left plots: Ventral‐to‐dorsal distribution. Right plots: Right‐to‐left distribution. [file NBM-38-e70053-s001.docx]

**Supplementary information**

**Specimen #1**


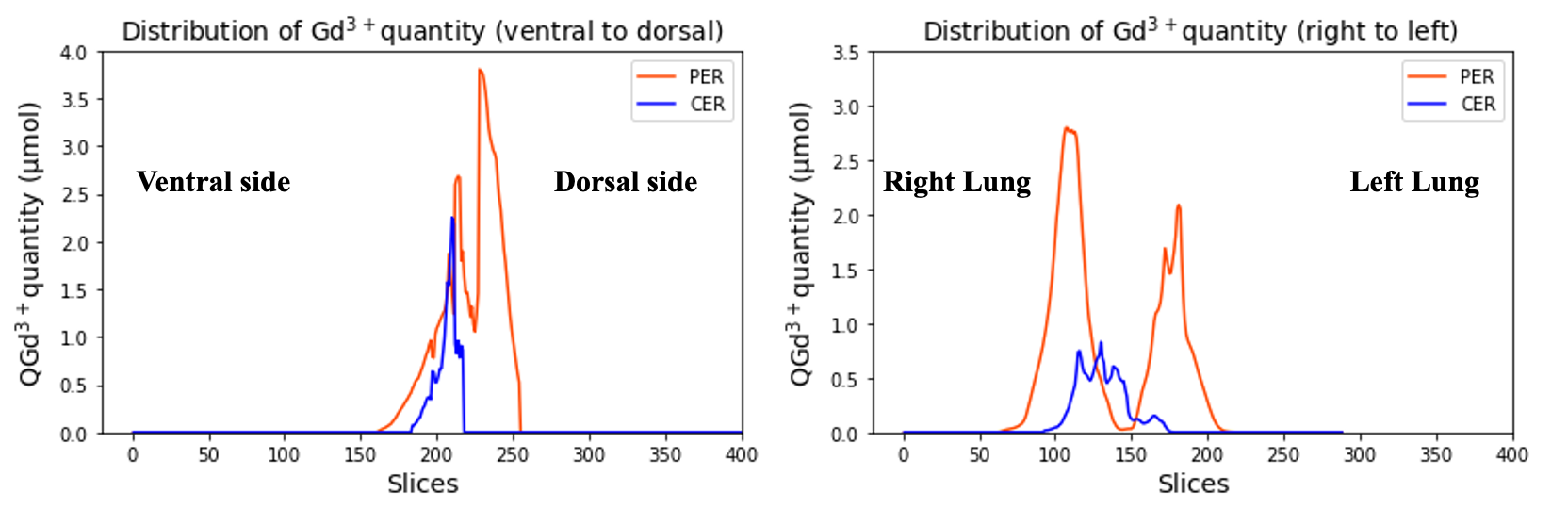


**Specimen #2**


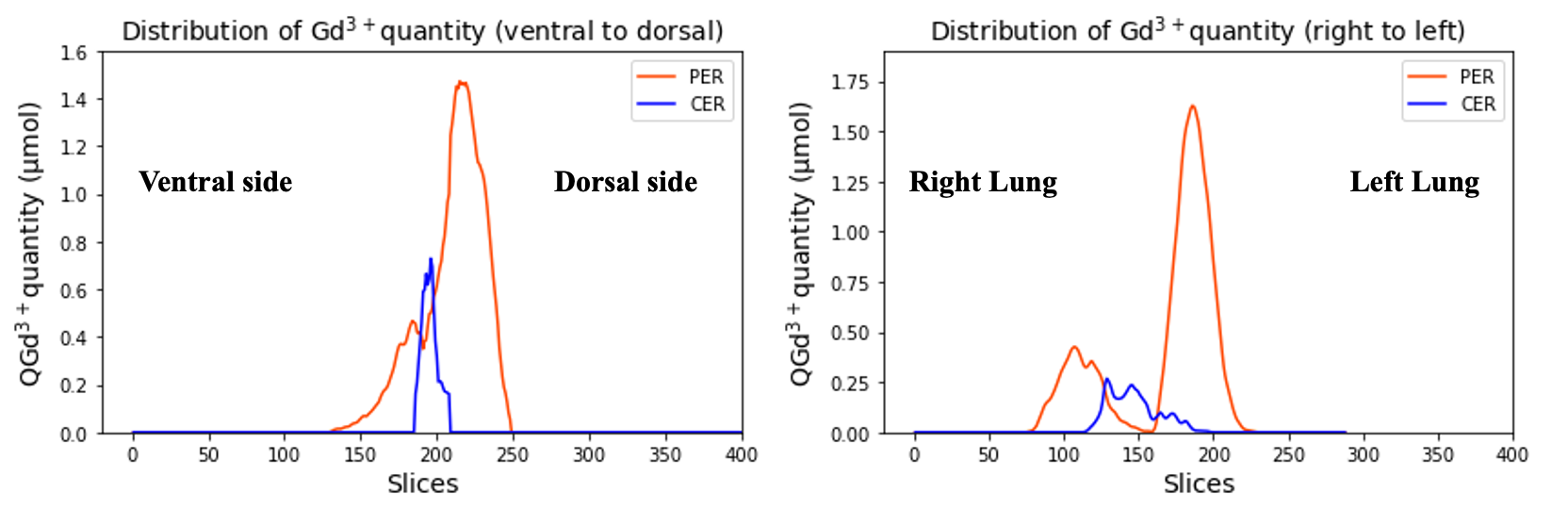


**Specimen #3**


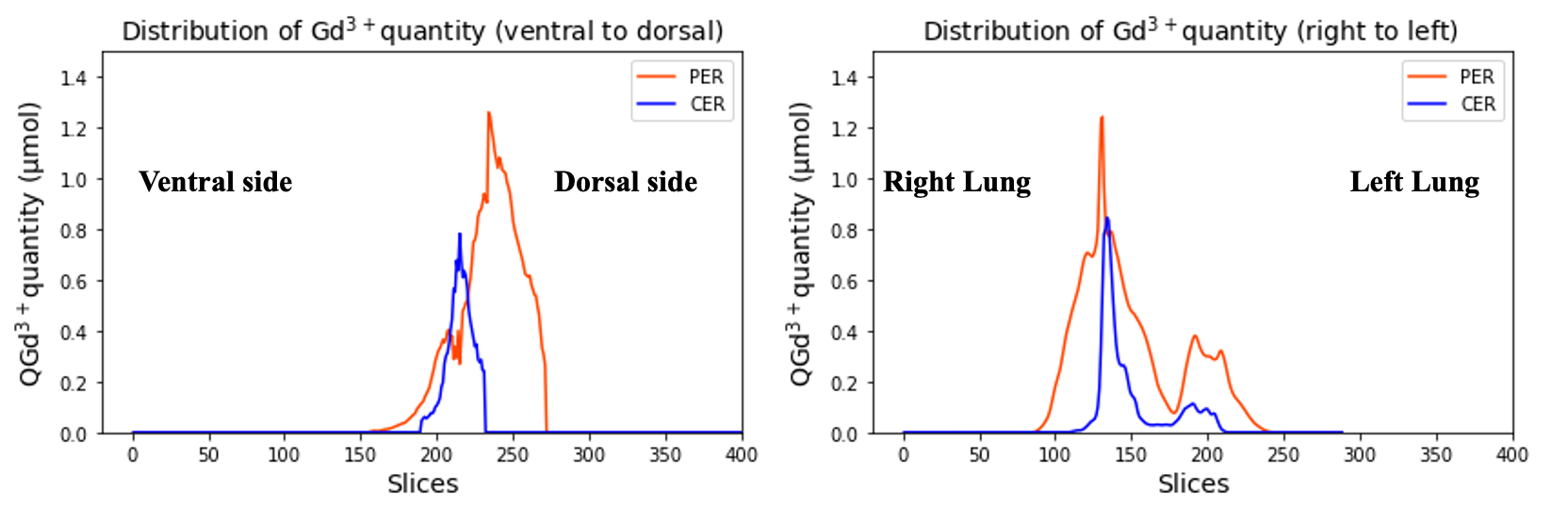


**Specimen #4**

**
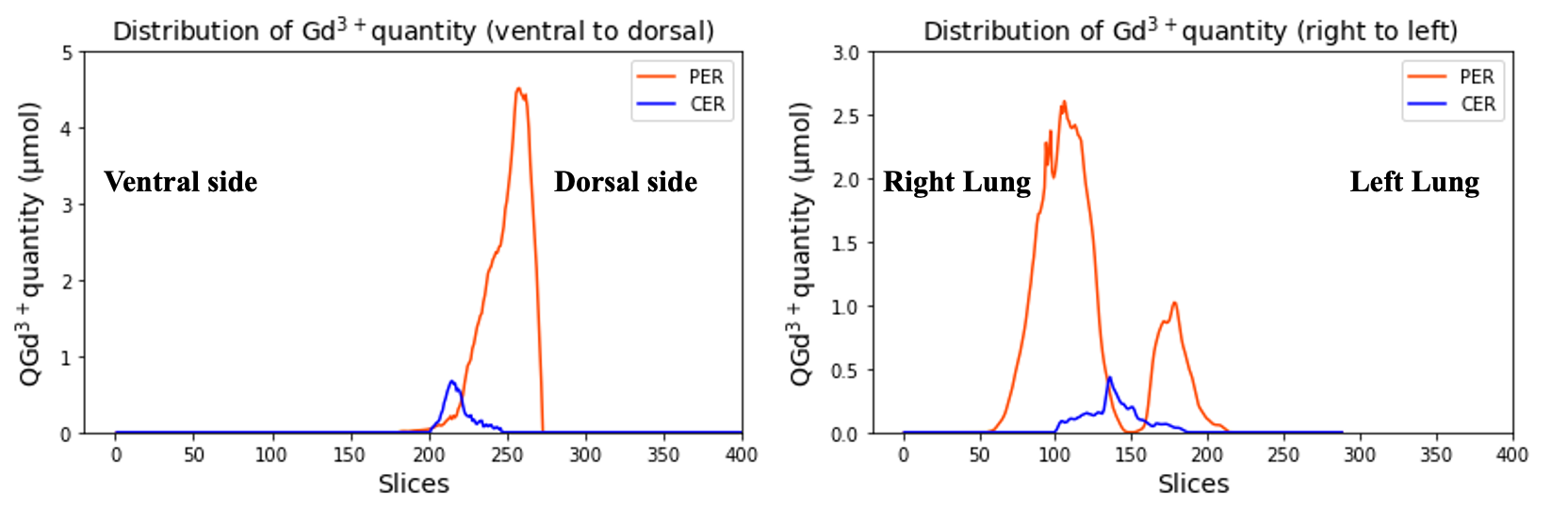
**

**Specimen #5**

**
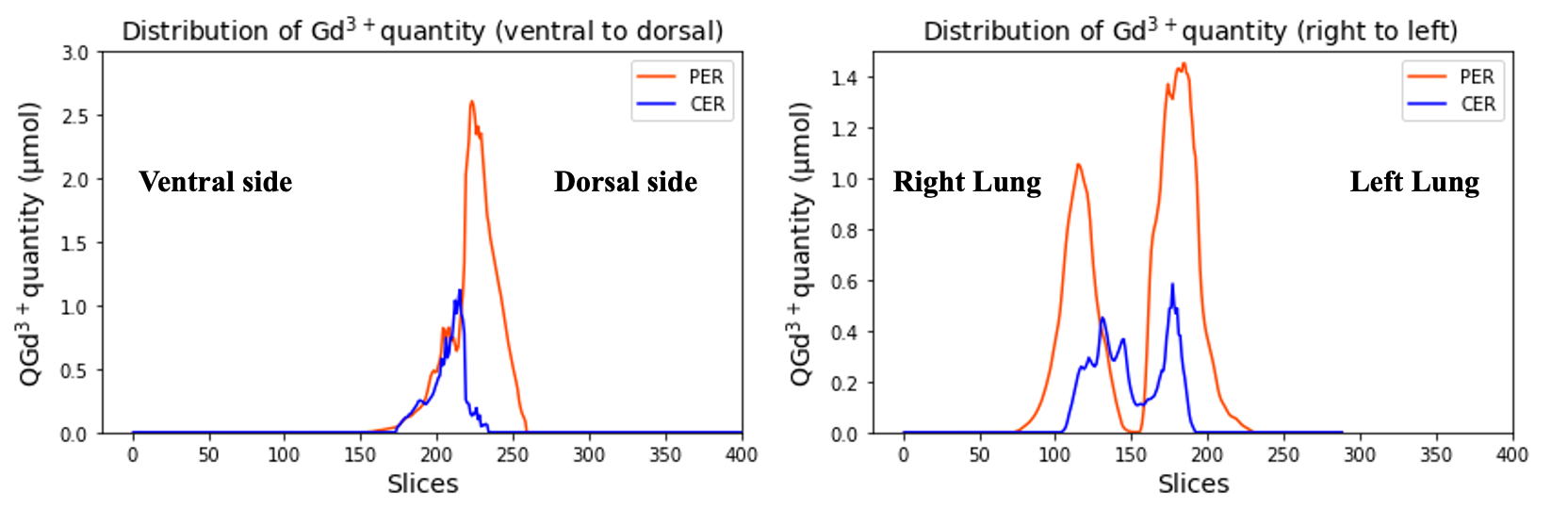
**

**Specimen #6**

**
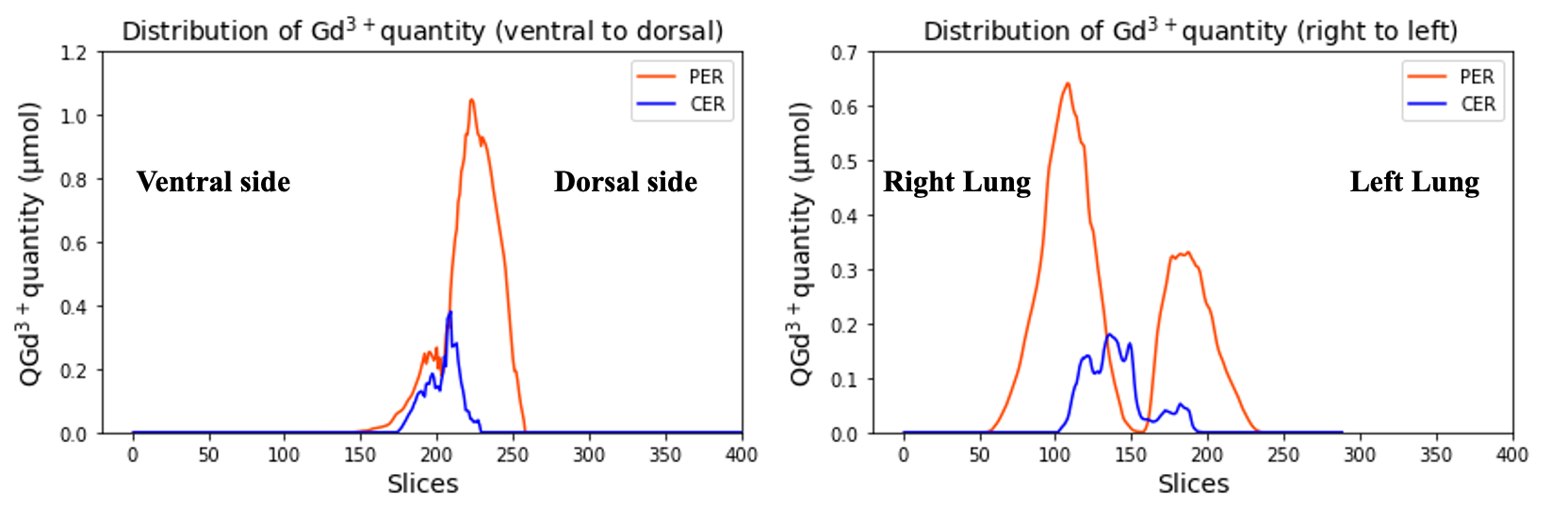
**

Fig. S1. Distribution of the chelated Gd^3+^ quantity (μmol) in the central enhanced regions (CER) and peripheral enhanced regions (PER) of the lungs for the six specimens of isolated thoraxes. Left plots: ventral to dorsal distribution. Right plots: right to left distribution.
